# Unravelling the efficacy and reproductive potential of newly discovered entomopathogenic nematodes, *Steinernema indicum* and *Steinernema shori* against *Spodoptera frugiperda* in maize

**DOI:** 10.3389/fpls.2026.1919468

**Published:** 2026-08-25

**Authors:** Jagadeesh Patil, Aarthi Nekkanti, Omprakash Navik, Manjunatha T. Gowda, Priyank Hanuman Mhatre, Kesavan Subaharan

**Affiliations:** 1 ICAR–National Bureau of Agricultural Insect Resources, Bengaluru, Karnataka, India; 2 ICAR–Indian Institute of Vegetable Research, Varanasi, Uttar Pradesh, India; 3 ICAR-Central Institute for Cotton Research, Nagpur, Maharashtra, India

**Keywords:** armyworm, maize, reproduction, *Steinernema* species, virulence

## Abstract

**Introduction:**

*Spodoptera frugiperda* (J.E. Smith), commonly known as the fall armyworm, is one of the most destructive insect pests of maize worldwide, causing substantial economic losses. Its widespread occurrence and increasing resistance to conventional insecticides have intensified the need for effective and sustainable management approaches. Reliance on chemical pesticides not only accelerates resistance development but also poses significant risks to the environment and beneficial non-target organisms. Consequently, environmentally friendly alternatives, particularly biological control strategies, are gaining importance as viable components of integrated pest management programs for this pest.

**Methods:**

In the present study, the susceptibility of the larval and pupal stages of *S. frugiperda* to two indigenous entomopathogenic nematode (EPN) species, *Steinernema shori* NBAIRS80 and *Steinernema indicum* NBAIRS58, was assessed. The effect of different concentrations of infective juveniles (IJs) of *S. indicum* and *S. shori* on their reproductive potential in third-instar *S. frugiperda* larvae was also investigated under laboratory conditions. Furthermore, the efficacy of *S. indicum* and *S. shori* against *S. frugiperda* was evaluated under both pot and field conditions.

**Results:**

Results revealed that under laboratory conditions, both EPN species exhibited high virulence against third, fourth, and sixth instar larvae, achieving 100.0% mortality as the concentration increased from 20 to 420 IJs larva^-1^. However, *S. shori* caused significantly higher mortality than *S. indicum*. A similar trend was recorded in pupae, where *S. shori* caused 81.0% mortality compared to 65.0% by *S. indicum*. Both species were able to penetrate the larvae and complete their life cycles, producing a higher number of infective juveniles (IJs). However, in both species, penetration declined significantly at higher IJ concentrations. Although emamectin benzoate performed better than both EPN species under both pot and field conditions, *S. shori* showed improved performance in reducing larval populations after the second round of spraying.

**Conclusion:**

Considering its effectiveness and environmental safety, the present study highlights the potential of *S. shori* as a biological control agent against the larval and pupal stages of *S. frugiperda. *Future studies should evaluate its efficacy against other economically important insect pests and assess its performance under diverse environmental conditions to support its wider field application.

## 1 Introduction

The fall armyworm (FAW), *Spodoptera frugiperda* (J. E. Smith), is an invasive species that poses a significant threat to maize and, consequently, to global food security (Stokstad, 2017). This pest spread from the neotropical regions of America to West Africa in early 2016 Goergen et al. (2016) and rapidly expanded to several countries across sub-Saharan Africa. In 2018, Karnataka, a state in India, reported its first FAW outbreak, which resulted in substantial economic losses in maize production (Shylesha et al., 2018; Sharanabasappa et al., 2018). Within a year, the pest had spread to major maize-growing areas of the country (Rakshit et al., 2019; Suby et al., 2020). This insect is highly polyphagous, capable of infesting more than 350 host plants, including maize (*Zea mays*), rice (*Oryza sativa*), sorghum (*Sorghum bicolor*), cotton (*Gossypium* spp.), and soybean (*Glycine max*) Barros et al. (2010). The larvae of FAW feed on both the vegetative and reproductive parts of plants, resulting in significant yield losses. As reported by Adhikari et al. (2020), *S. frugiperda* infestations can cause 50-80% damage to maize yields, leading to financial losses amounting to millions of dollars. In 2017, India’s maize production reached 28.7 million tonnes; however, a decline of 3.8% was recorded due to fall armyworm infestation, resulting in total production of 27.8 million tonnes Singh et al. (2023). In addition, the cost of managing this pest with chemical insecticides in maize was estimated at US$71.23, US$64.48, and US$56.01 per hectare during 2018, 2019, and 2020, respectively (Sharanabasappa et al., 2021). Given its economic importance and the growing concern over resistance to chemical insecticides, there is an urgent need for alternative and sustainable management strategies for this pest.

In this context, biological control agents can play an important role in managing insect pests and increasing food production in a more sustainable manner Chaudhary et al. (2024). Among various biocontrol agents, entomopathogenic nematodes (EPNs) are recognized as beneficial nematodes that have attracted significant interest as biological control agents for effectively managing a range of insect pests. Nematodes primarily belonging to the families Steinernematidae and Heterorhabditidae are highly effective in managing numerous subterranean or concealed pests, as well as certain aboveground insect pests (Shapiro-Ilan and Lewis, 2024). The free-living, non-feeding third-stage juveniles of *Steinernema* and *Heterorhabditis* are known as infective juveniles (IJs). *Steinernema* IJs have a symbiotic relationship with *Xenorhabdus*, whereas *Heterorhabditis* IJs are associated with *Photorhabdus*, both of which inhabit their intestines Boemare (2002) and Tarasco et al. (2023). Typically, *Steinernema* IJs enter the insect haemocoel through natural openings such as the mouth, anus, and spiracles. In contrast, *Heterorhabditis* IJs can penetrate both natural openings and the intersegmental membranes of the cuticle. After entry, the IJs release their symbiotic bacteria into the haemocoel. The bacteria proliferate in the insect haemolymph, leading to host death, usually within 24 to 48 hours. Following host death, the nematodes continue feeding on the host tissues, developing and reproducing. The juveniles undergo four developmental stages before reaching maturity. Depending on resource availability, one or more generations may develop within the host cadaver, resulting in a large number of IJs being released into the environment to infect additional hosts and sustain their life cycle (Kaya and Gaugler, 1993). The use of EPNs for the management of insect pests has become increasingly popular due to their broad host range, host-searching ability, and efficiency in infecting insect pests found both above and below ground. The efficacy of various EPNs against *S. frugiperda* has been documented in earlier studies, including *S. riobrave*, *H. indica*, *S. arenarium*, *Heterorhabditis* sp., *S. carpocapsae*, and *S. longicaudum*, highlighting their potential as biological control agents (Molina-Ochoa et al., 1999; Garcia et al., 2008; Andalo et al., 2010; Acharya et al., 2020; Patil et al., 2022; Ratnakala et al., 2023).

However, numerous studies have indicated that the virulence of native strains or species is often greater than that of non-native species or strains. Therefore, the application of indigenous EPN species for pest control is of utmost importance. This study aims to evaluate the efficacy of two newly discovered EPN species, *S. indicum* NBAIRS58, isolated from the soil of a coconut palm (*Cocos nucifera*) in the Udupi district of Karnataka, India, and *S. shori* NBAIRS80, isolated from the rhizosphere of sal (*Shorea robusta*) in the Bastar district of Chhattisgarh, India, using the soil baiting technique (Bedding and Akhurst, 1975). Since these two EPN species are native to India and newly described to science, it is crucial to evaluate their virulence and reproductive potential against insect pests before they are widely used in pest management programs. This assessment is necessary to confirm that they serve as effective, safe, and sustainable alternatives to chemical insecticides. Thus, the objectives of this study are to (1) assess the virulence of the newly discovered *S. indicum* and *S. shori* against both larvae and pupae of *S. frugiperda*, (2) investigate the reproductive potential of *S. indicum* and *S. shori* in larvae of *S. frugiperda*, and (3) evaluate the efficacy of *S. indicum* and *S. shori* against *S. frugiperda* under pot and field conditions.

## 2 Methodology

### 2.1 Source of insects

Larvae of *S. frugiperda* were initially collected from untreated maize fields showing natural infestation, ensuring that no chemical insecticides or biopesticides had been applied. Each larva was reared separately on a semi-synthetic diet (Prasanna et al., 2018). until pupation. The resulting pupae were then placed in Petri dishes lined with moist filter paper and kept inside insect-rearing cages. To support adult emergence and survival, a sterile cotton ball soaked in a 10% honey solution was provided as a food source. Butter paper sheets were introduced into the cages to serve as a substrate for egg laying. After oviposition, eggs were carefully collected and transferred into glass tubes (2 cm × 8 cm). Once the eggs hatched, the neonate larvae were moved to multicellular rearing trays (Ballal et al., 1998). containing the same semi-synthetic diet. Both larval and pupal stages were later used for evaluating their susceptibility to EPNs. Additionally, colonies of the greater wax moth, *Galleria mellonella* (L.), were maintained on a modified artificial diet following previously established methods (Patil et al., 2020a).

### 2.2 Source of nematodes

*Steinernema indicum* NBAIRS58 and *S. shori* NBAIRS80, originally isolated by Patil et al. (2023) and Soni et al. (2023), respectively, from India, were maintained live at the Division of Germplasm Collection and Characterization, ICAR-National Bureau of Agricultural Insect Resources, Bengaluru, Karnataka, India. Both EPN species were cultured on *G. mellonella* according to the methodology outlined by Patil et al. (2020a). Wax moth larvae were inoculated with 25 IJs per larva in Petri dishes lined with a single layer of Whatman No. 1 filter paper. Next, 1 mL of distilled water containing 100 IJs was applied to each dish, after which four last-instar larvae of *G. mellonella* were introduced. The dishes were maintained at 23 ± 2 °C for incubation. Once the larvae died, their cadavers were transferred to White traps (Kaya and Stock, 1997) and checked daily for IJ emergence. The newly emerged IJs were collected and stored in tissue culture flasks at 15 °C. Only IJs that were one week old were used for subsequent experiments.

### 2.3 Laboratory experiments

#### 2.3.1 Virulence of EPNs against 3^rd^ and 4^th^ instars of *S. frugiperda*

This assay was performed using plastic Petri dishes with a diameter of 9 cm. Each dish was lined with a single layer of Whatman No. 1 filter paper. The Petri dish lids were perforated with two to three small holes to provide sufficient ventilation for the insects. A one-day-old single third- or fourth-instar larva was placed individually into each dish. During the experiment, 3.0 g semi-synthetic diet was placed in the Petri dishes to serve as food for the larvae. The insects were subsequently allowed to settle in the Petri dishes for 24 hours. After 24 hours, each larva was inoculated with either *S. indicum* or *S. shori* at concentrations of 20, 40, 80, 160, 320, or 420 IJs larva^-1^. Control larvae received 1 mL of distilled water without IJs. These concentrations were selected based on the preliminary experiment results. The dishes were then incubated at 24 ± 1 °C with 16:8 h L:D, and larval mortality was recorded daily for five days. To confirm that larval death was attributable to EPN infection, cadavers were individually dissected in Ringer’s solution under a dissecting microscope; only those insects in which nematodes were observed were considered to have been killed by EPNs. Each treatment included five replicates, with ten Petri dishes per replicate, and the experiment was conducted twice. The experimental design was completely randomized.

#### 2.3.2 Soil column assay to test the virulence of EPNs against 6^th^ instar of *S. frugiperda*

The fully grown sixth-instar larvae of *S. frugiperda* leave the plants and enter the soil at a depth ranging from 2.0 to 8.0 cm, where they construct a protective chamber using soil particles and silk to form a cocoon for pupation. Therefore, in this assay, soil comprising 72.0% sand, 12.0% silt, 10.0% clay, and 2.0% organic matter, with a pH of 6.4 and 13.0% moisture content, was placed in polyvinyl chloride (PVC) tubes (12.0 cm in height with an internal diameter of 2.8 cm) and filled to a depth of 10.0 cm. A one-day-old one sixth-instar larva of *S. frugiperda* was buried at a depth of 8.0 to 9.0 cm. Subsequently, *S. indicum* or *S. shori* at concentrations of 20, 40, 80, 160, 320, or 420 IJs larva^-1^ was applied to the surface of the tubes. Tubes containing distilled water without IJs served as the control treatment. The tubes were maintained in a vertical position and incubated at 23.0 ± 2 °C. After five days, the tubes were disassembled, and larval mortality was confirmed by dissecting the cadavers in Ringer’s solution under a dissecting microscope; only cadavers in which nematodes were observed were considered to have been killed by EPNs. Each treatment consisted of five replicates, with 10 tubes per replicate, and the experiment was conducted twice. The experimental design was completely randomized.

#### 2.3.3 Virulence of EPNs against pupal stage of *S. frugiperda*

A standardized procedure was followed for inoculating the pupae of *S. frugiperda* with IJs of either *S. indicum* or *S. shori* (Patil et al., 2020b). Plastic containers with a diameter of 4.8 cm, a height of 5 cm, and a surface area of 18.06 cm^2^ were used for this assay. The containers were filled with 60.0 g of soil (comprising 72.0% sand, 12.0% silt, 10.0% clay, and 2.0% organic matter, with a pH of 6.4) adjusted to 13.0% moisture content. A two-day-old single pupa of *S. frugiperda* was placed in each container at a depth of 1.5 cm and covered with soil. Subsequently, either *S. indicum* or *S. shori* was inoculated onto the soil surface of each container at doses of 50, 100, 300, 400, or 500 IJs pupa^-1^. Containers treated with 3 mL of distilled water served as the control and were maintained at 23.0 ± 2 °C. The containers were then examined daily for up to ten days, and the percentage of adult eclosion was recorded. Non-emerged pupae were considered dead if nematodes were found within their bodies upon dissection under a stereozoom microscope. Adult moths that emerged were transferred to a 9 cm Petri dish lined with two layers of Whatman No. 1 filter paper, where their mortality was monitored. After death, adult moths were dissected in Ringer’s solution under a dissecting microscope. Each treatment included five replicates, with ten containers per replicate, and the experiment was conducted twice. The experimental design was completely randomized.

#### 2.3.4 Reproductive potential of *S. indicum* and *S. shori* in *S. frugiperda*

To investigate the effect of varying concentrations of IJs of *S. indicum* and *S. shori* on their penetration, third-instar larvae of *S. frugiperda* were examined using 9 cm Petri dishes. Each dish was lined with a single layer of Whatman No. 1 filter paper, and a single third-instar larva was placed in each dish. Subsequently, the larvae were treated with either *S. indicum* or *S. shori* at concentrations of 100, 200, 300, 400, or 500 IJs larva^-1^. After the death of *S. frugiperda*, the larvae were rinsed with water to remove any IJs adhering to their surfaces. The cadavers were individually dissected in Ringer’s solution, then examined under a dissecting microscope to determine the number of IJs that had penetrated each larva. Each treatment included ten replicates with ten dishes per replicate, and the experiment was conducted twice.

In the reproduction assay, third-instar larvae of *S. frugiperda* were treated like the penetration assay using 9 cm Petri dishes. A single larva was placed in each dish and infected with either *S. indicum* or *S. shori* at a density of 200 or 300 IJs larva^-1^. Once the larvae died, the cadavers were transferred to White traps, which were subsequently incubated at 23 ± 2 °C. Seven days post-incubation, IJs emerging from the cadavers were collected and expressed as IJs per milligram of *S. frugiperda* larval body weight. Each treatment included ten replicates with ten dishes per replicate, and the experiment was conducted twice.

### 2.4 Pot experiment

In this experiment, the efficacy of *S. indicum* and *S. shori* against fourth-instar *S. frugiperda* was evaluated. Plastic pots (25.5 cm in diameter and 22.5 cm in depth) were filled with 6,000 g of autoclaved soil (72.0% sand, 12.0% silt, 10.0% clay, and 2.0% organic matter, with a pH of 6.4) adjusted to 13.0% moisture content. Two seeds of corn (Hybrid Sweet 70) were sown, and the pots were watered once a week throughout the experiment. Plants were supplied with the recommended dose of fertilizers. Twenty-five days after sowing (V6 stage, knee-high stage of the crop), the average plant height and number of leaves at the V6 stage were 58.6 ± 1.9.6 cm and 6 leaves plant^-1^, respectively. Four fourth-instar *S. frugiperda* larvae were introduced into the whorl of each plant. They were initially allowed to establish and feed on the plants for two days. Two days after, the plants were treated with either *S. indicum* or *S. shori* at doses of 500 or 1, 000 IJs larva^-1^ (approximately 2000 or 4, 000 IJs plant^-1^). In addition to the EPN treatments, emamectin benzoate (Fitrest 5 SG soluble granules at 12.5 g a.i. ha^-1^) was used as a positive control, and water was used as a negative control. The pots were then maintained at 26.0 ± 1 °C, with a photoperiod of 16:8 h (L:D) and 70.0% RH. Every day, 4 mL of water was sprayed with a hand sprayer to onto the whorl to simulate the conditions of natural dew. The experiment was arranged in a completely randomized design, and each treatment consisted of ten replicate pots. Larval mortality was recorded five days post-treatment using destructive sampling. Dead larvae were dissected under a dissecting microscope to confirm whether mortality was due to EPN infection.

### 2.5 Field experiment

A field experiment was carried out in a maize field naturally infested with *S. frugiperda* at Bommanahalli village (13°38′ N, 77°44′ E) in Chikkaballapura taluk and district, Karnataka, India, during the Rabi season. The study consisted of 40 experimental plots, each measuring 8 × 5 m (40 m²), with a spacing of 2 m between plots. Four treatments were evaluated; each replicated ten times and arranged in a completely randomized block design. Adjacent plots were separated by three untreated rows of plants that served as buffer rows. Maize seeds (Hybrid Sweet 70) were sown during mid-November 2025 at an inter-row and intra-row spacing of 0.6 m and 0.2 m, respectively, resulting in a plant population of 55, 555 plants ha^−1^. Treatments were suspended in water and uniformly applied using a spray volume of 400 L ha^−1^. A sprayer fitted with a flat-fan nozzle and operated at 300 kPa ensured uniform application. Applications were performed between 4:00 PM and 5:00 PM to reduce UV exposure to EPNs. During application, the temperature was approximately 25.0 °C with relative humidity ranging from 70.0% to 80.0%. The treatments included *S. shori* and *S. indicum* applied at a rate of 2.5 × 10^8^ ha^-1^, and emamectin benzoate (Fitrest 5 SG, Bayer Crop Science Pvt. Ltd., Thane, Maharashtra) at 12.5 g a.i. ha^-1^, along with an untreated control. Control plots received only water. All recommended agronomic practices for maize cultivation were followed. Treatments were applied twice during the vegetative stage of the crop: first at 20 days after sowing (V4 stage, weaning stage) and again at 40 days after sowing (V8 stage, knee-high stage). The average plant height and number of leaves at the V4 stage were 31.4 ± 2.6 cm and 4 leaves plant^-1^, respectively, while at the V8 stage they were 73.2 ± 4.2 cm and 8 leaves plant^-1^, respectively. Pre- and post-spraying viability of IJs was 99 and 98%, respectively. For data collection, 200 consecutive maize plants were selected per treatment at each stage. Observations were recorded before spraying, after the first spray, and after the second spray. Non-destructive sampling was followed to count the number of larvae. Counts were expressed as the number of larvae per 20 plants (Patil et al., 2022).

### 2.6 Statistical analysis

Mortality data for *Spodoptera frugiperda* larvae and pupae were corrected using Abbott’s formula (Abbott, 1925) prior to analysis. No significant variation (α > 0.05) was detected between experimental repeats; therefore, the data were combined for further analysis. The larval bioassay data were subjected to three-way generalized linear mixed models (GLMM) implemented in PROC GLIMMIX, assuming a binomial distribution with a logit link function, considering IJ concentration, EPN species, and larval instar as the fixed factors. Data obtained from the pupal bioassay and soil column experiments were analysed using two-way GLMM with binomial distribution, logit link function, with IJ concentration and EPN species as the fixed factors. Pot experiment data were analysed by one-way GLMM with binomial distribution, logit link function. Field experiment larval count data were analysed using a generalized linear model (GLM) with a Poisson distribution and log link function. In contrast, IJ penetration and reproduction in third-instar larvae of *S. frugiperda* were analyzed using one-way ANOVA. Differences among treatment means were determined using Tukey’s *post hoc* test at a significance level of α = 0.05, followed by Tukey’s *post hoc* test (α < 0.05) for mean separation. All analyses were performed using SAS software (version 9.3, 2011; SAS Institute, Cary, NC, USA) (SAS Institute Inc, 2011).

## 3 Results

### 3.1 Virulence of EPNs against 3^rd^ and 4^th^ instars of *S. frugiperda*

**Figure 1:**
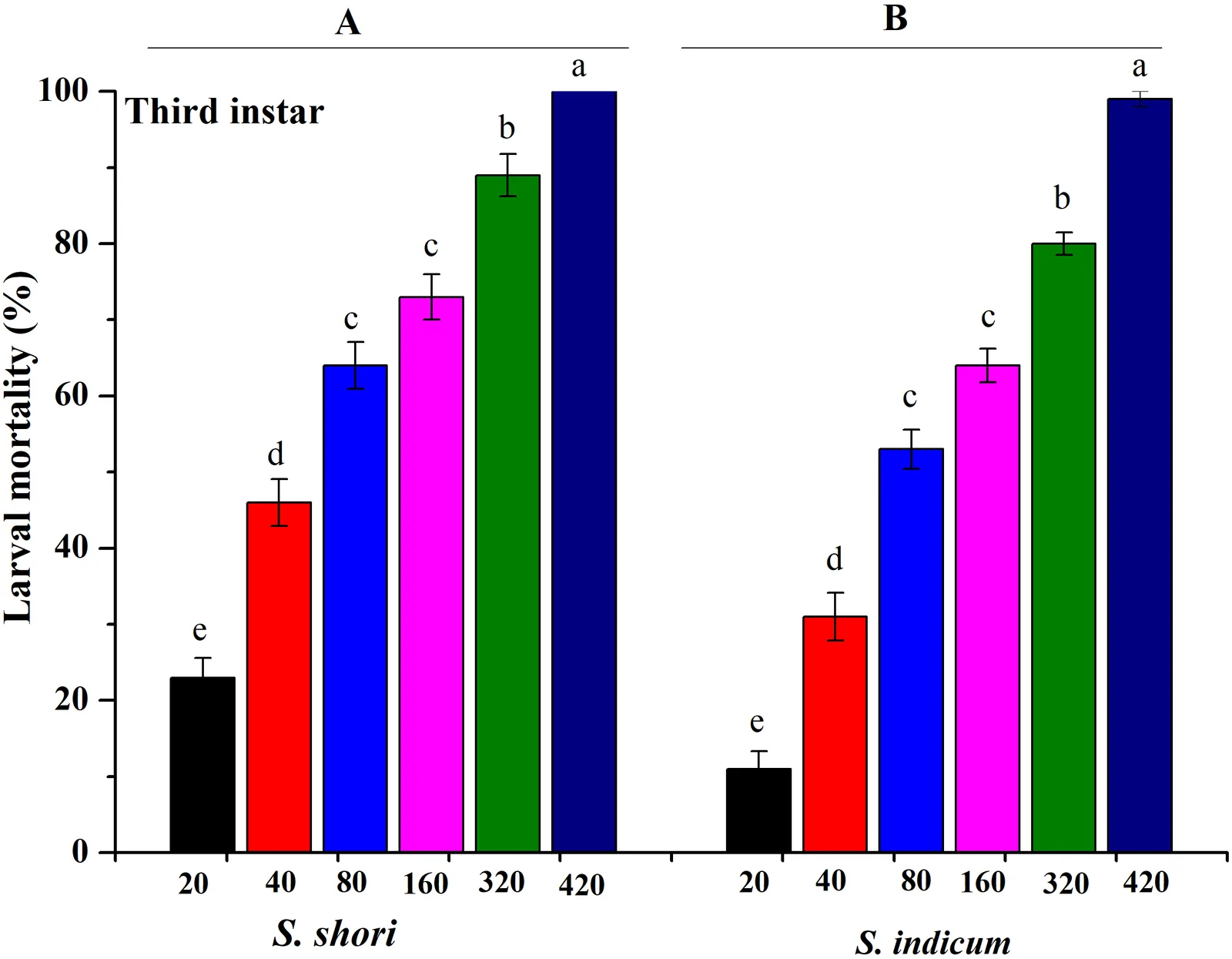
Corrected mortality (mean % ± SE) of third-instar larvae of *Spodoptera frugiperda* exposed to different concentrations of *Steinernema shori* NBAIRS80 and *S. indicum* NBAIRS58 after five days of treatment under laboratory conditions. Bars with different lower-case alphabets on top of the error bars depict statistically significant differences for IJs concentrations, and alphabets with upper-case letters denote significant differences between the nematode species (P < 0.05, Tukey’s test) (n = 100).

**Figure 2:**
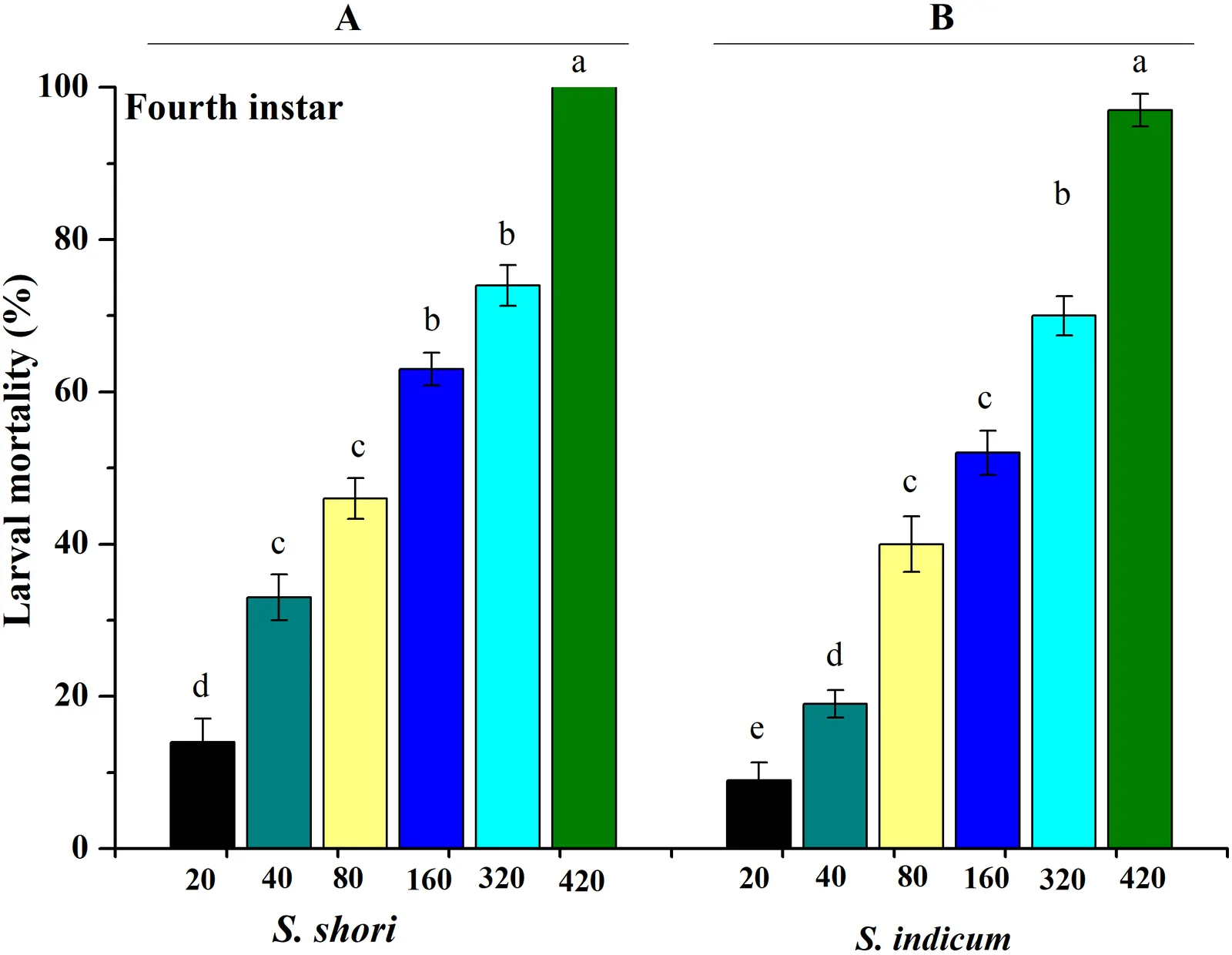
Corrected mortality (mean % ± SE) of fourth-instar larvae of *Spodoptera frugiperda* exposed to different concentrations of *Steinernema shori* NBAIRS80 and *S. indicum* NBAIRS58 after five days of treatment under laboratory conditions. Bars with different lower-case alphabets on top of the error bars depict statistically significant differences for IJs concentrations, and alphabets with upper-case letters denote significant differences between the nematode species (P < 0.05, Tukey's test) (n = 100).

The results of this assay showed that *S. shori* NBAIRS80 caused a 23.0% mortality in third instar larvae of *S. frugiperda* when exposed to 20 IJs larva^−1.^ However, this mortality escalated to 100.0% with 420 IJs larva^−1^ (F_5, 45_ = 17.74; P < 0.0001) (**Figure 1**). Similarly, for fourth-instar larvae, *S. shori* NBAIRS80 induced only a 14.0% mortality rate at 20 IJs larva^−1^, but this rate rose to 100.0% when the concentration was increased to 420 IJs larva^−1^ (F_5, 45_ = 15.68; P < 0.0001). Conversely, *S. indicum* NBAISS58 caused 11.0% and 9.0% mortality in the third (F_5, 45_ = 23.23; P < 0.0001) and fourth instars (F_5, 45_ = 25.42; P < 0.0001) larvae of *S. frugiperda* at a dosage of 20 IJs larva^−1^, respectively, with mortality significantly rising to 100.0% as the dosage increased to 420 IJs larva^−1^ (**Figure 2**). A three-way generalized linear mixed model demonstrated that the mortality attributed to *S. shori* NBAIRS80 was significantly greater than that caused by *S. indicum* NBAIRS58 in both the third and fourth instars (F_1, 207_ = 65.18; P < 0.0001), with third instar larvae exhibiting greater susceptibility than fourth instar larvae (F_1, 207_ = 7.89; P < 0.0001). Notably, a significant interaction solely between the concentrations of IJs and the instars *S. frugiperda* (F_5, 207_ = 1.45; P = 0.031) was recorded.

### 3.2 Soil column assay to test the virulence of EPNs against 6^th^ instar of *S. frugiperda*

**Figure 3:**
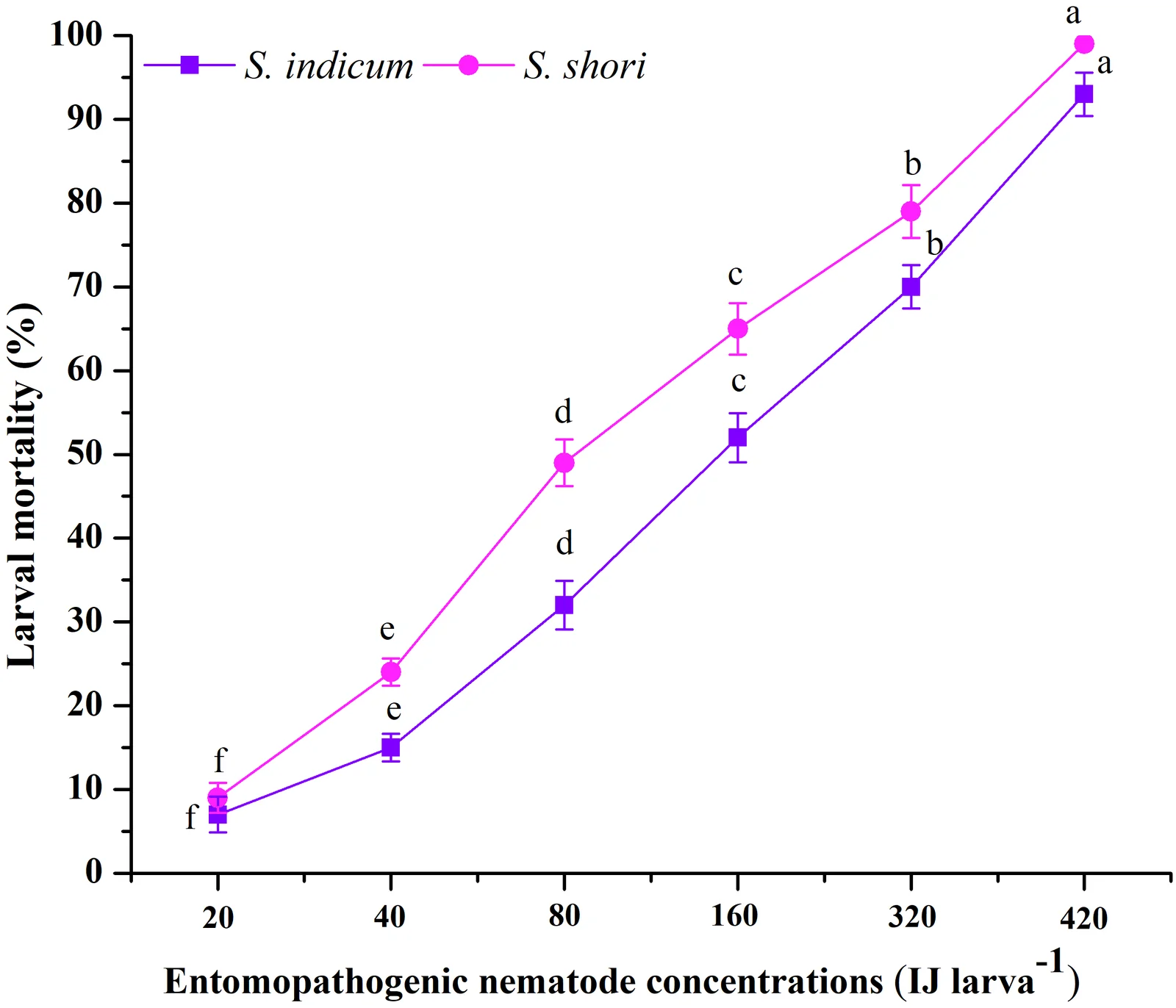
Corrected mortality (mean % ± SE) of sixth-instar larvae of *Spodoptera frugiperda* in a soil column assay. The bioassay was conducted in polyvinyl chloride (PVC) tubes with a single larva in each tube under laboratory conditions. Different concentrations of either *S. indicum* NBAIRS58 or *S. shori* NBAIRS80 were applied to the surface of the tubes. Bars with different alphabets on top of the error bars depict statistically significant differences for IJs concentrations (P < 0.05, Tukey's test) (n = 100).

In this soil column assay, a mortality rate of only 9.0% when sixth instar *S. frugiperda* larvae were treated with 25 IJs larva^−1^, and a mortality rate of 7.0% with *S. indicum* NBAIRS58 under the same treatment of 25 IJs larva^−1^ was recorded. However, when the concentration was raised to 420 IJs larva^−1^, the mortality attributed to *S. shori* NBAIRS80 dramatically increased to 100.0% (F_5, 45_ = 21.23; P < 0.0001), while the mortality associated with *S. indicum* NBAIRS58 rose significantly to 93.0% (F_5, 45_ = 29.90; P < 0.0001). As the concentration increased, the mortality rates caused by both nematode species showed a significant rise (**Figure 3**). A three-way generalized linear mixed model indicated that the mortality caused by *S. shori* NBAIRS80 was significantly greater than that caused by *S. indicum* NBAIRS58 (F_1, 99_ = 15.44; P < 0.0001). A significant interaction was detected between the concentrations of IJs and the nematode species (F_6, 99_ = 2.4; P = 0.032).

### 3.3 Virulence of EPNs against pupal stage of *S. frugiperda*

**Figure 4:**
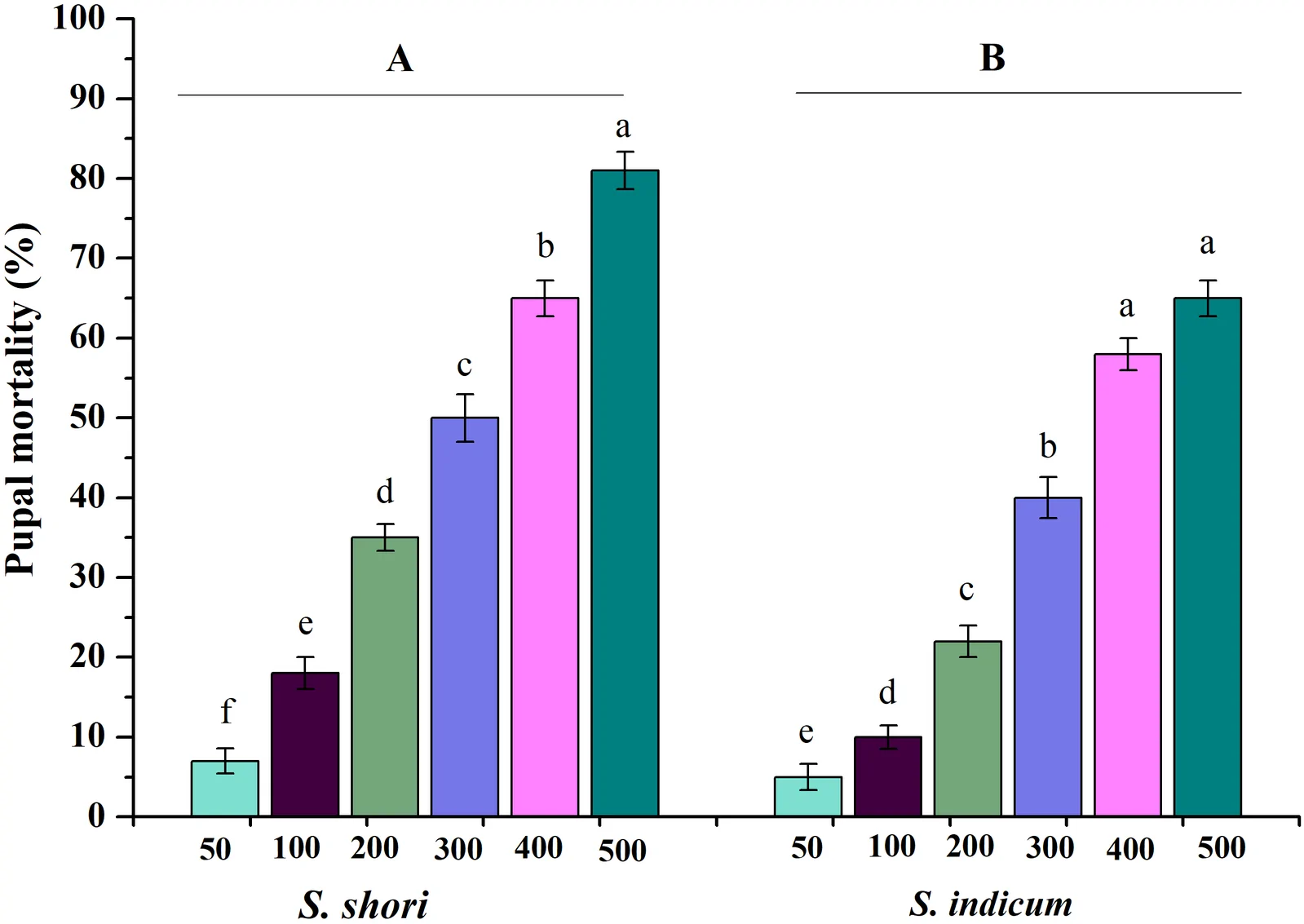
Corrected mortality (mean % ± SE) of pupae of *Spodoptera frugiperda* exposed to different concentrations of *Steinernema shorii* NBAIRS80 and *S. indicum* NBAIRS58 after 10 days of treatment under laboratory conditions. Bars with different lowercase letters atop the error bars depict statistically significant differences for IJs concentrations, while letters with uppercase denote significant differences between the nematode species (P < 0.05, Tukey's test) (n = 100).

Both nematode species are able to cause mortality in the pupae of *S. frugiperda*, but mortality significantly varied with IJs concentrations (F_5, 99_ = 45.88; P < 0.0001). When pupae were inoculated with *S. shori* NBAIRS80 at a lower concentration of 50 IJs pupa^−1^, recorded only 7.0% mortality, and at a higher concentration of 500 IJs pupa^−1^, 81.0% mortality was recorded (F_5, 45_ = 24.99 P < 0.0001). Similarly, *S. indicum* NBAIRS58 caused 5.0% pupal mortality at a concentration of 50 IJs pupa^−1^, which increased substantially to 65.0% at 500 IJs pupa^−1^ (F_5, 45_ = 21.48 P < 0.0001) (**Figure 4**). The mortality caused by *S. shori* NBAIRS80 was significantly higher than that caused by *S. indicum* NBAIRS58 (F_1, 99_ = 11.59; P = 0.001). No significant interaction was recorded between IJs concentrations and nematode species.

### 3.4 Reproductive potential of *S. indicum* and *S. shori* in *S. frugiperda*

**Figure 5:**
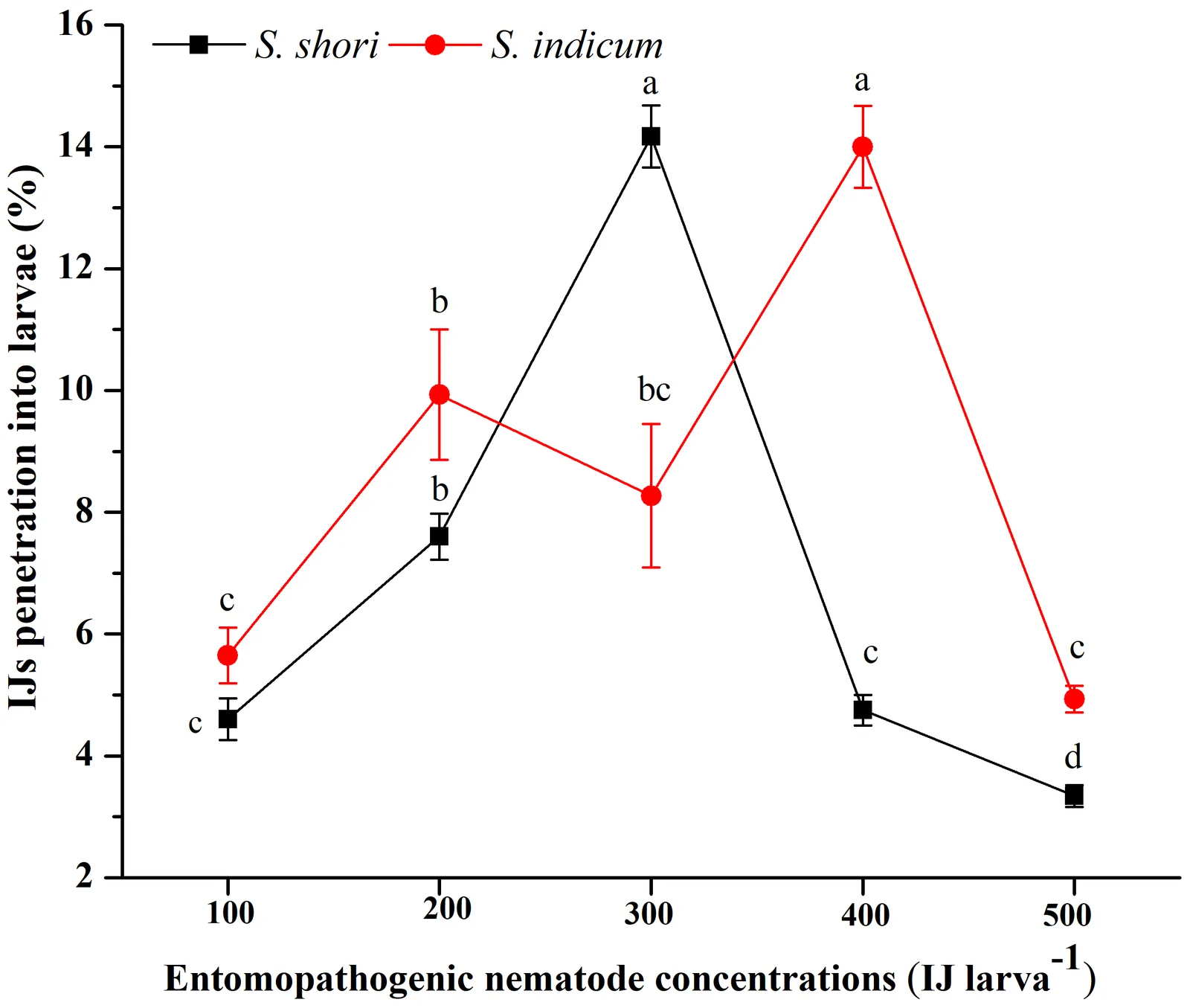
Mean percentage ± SE penetration of infective juveniles of *Steinernema shori* NBAIRS80 and *S. indicum* NBAIRS58 into the third instar larvae of *Spodoptera frugiperda* at various concentrations under laboratory conditions. Bars with different lower-case alphabets on top of the error bars depict statistically significant differences for IJs concentrations (P < 0.05, Tukey’s test) (n = 40).

Both nematode species successfully entered the haemocoel of third-instar *S. frugiperda* larvae and completed their reproduction. The level of penetration differed significantly between the species (F_1, 190_ = 18.61; P < 0.0001). For *S. shori* NBAIRS80, penetration was 4.6% at an inoculation dose of 100 IJs larva^−1^ and increased to 14.2% when the dose was raised to 300 IJs larva^−1^. However, the percent penetration significantly decreased when larvae were treated with higher concentrations of 400 or 500 IJs larva^−1^ (F_4, 95_ = 134.06; P < 0.0001). For *S. indicum* NBAIRS58, the percent penetration was 5.7% with 100 IJs larva^−1^, and the highest penetration rate was noted at 400 IJs larva^−1^, but it significantly dropped to 4.9% at 500 IJs larva^−1^ (F_4, 95_ = 19.74; P < 0.0001) (**Figure 5**). A two-way ANOVA demonstrated a significant difference between the concentrations of IJs and the nematode species (F_4, 190_ = 36.10; P < 0.0001).

**Figure 6:**
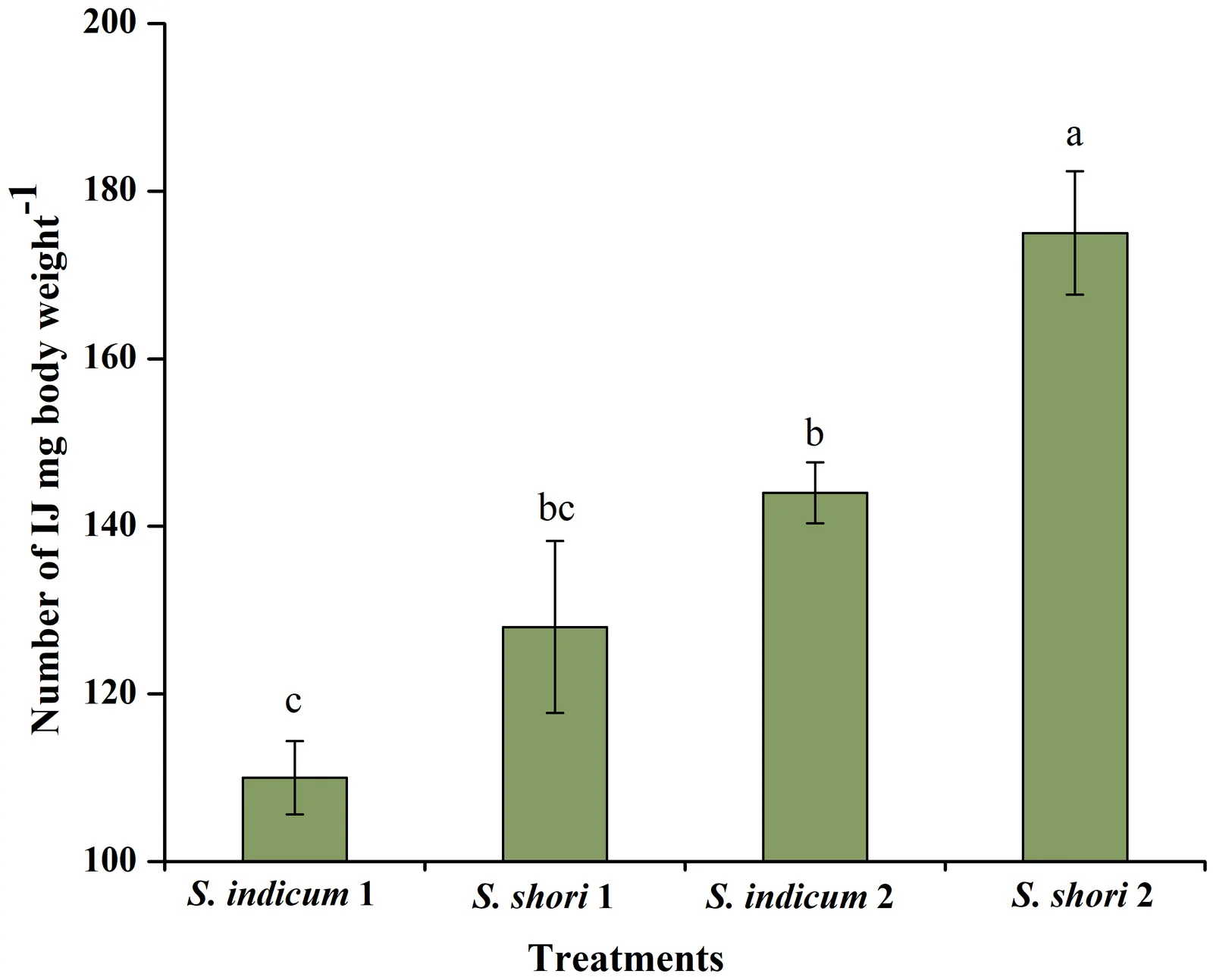
Multiplication of infective juveniles (per milligram of body weight of the host ± SE) of *Steinernema shori* NBAIRS80 and *S. indicum* NBAIRS58 in third-instar larvae of *Spodoptera frugiperda*. Each third-instar larva of *S. frugiperda* was inoculated with different concentrations of *S. shori* NBAIRS80 or *S. indicum* NBAIRS58. 1 = 200 IJs larva^-1^ and 2 = 300 IJs larva^-1^. Bars with different letters on top of error bars depict statistically significant differences between the treatments (P < 0.05, Tukey’s test) (n = 40).

*Steinernema shori* NBAIRS80 yielded 128 IJs mg^−1^ of host body weight in third-instar larvae when applied at 200 IJs larva^−1^, and this increased to 175 IJs mg^−1^ at a dose of 300 IJs larva^−1^. In contrast, *S. indicum* NBAIRS58 produced 90 IJs mg^−1^ body weight at 100 IJs larva^−1^ and 159 IJs mg^-1^ at 300 IJs larva^−1^. For both species, increasing the IJ concentration to 300 IJs larva^−1^ resulted in a significant rise in multiplication (F_1, 76_ = 28.40; P < 0.0001) (**Figure 6**). Overall, *S. shori* NBAIRS80 exhibited a significantly greater multiplication rate than *S. indicum* NBAIRS58 (F_1, 76_ = 28.40; P = 0.0104). However, the interaction between nematode species and IJ concentration was not statistically significant according to the two-way ANOVA.

### 3.5 Pot experiment

**Figure 7:**
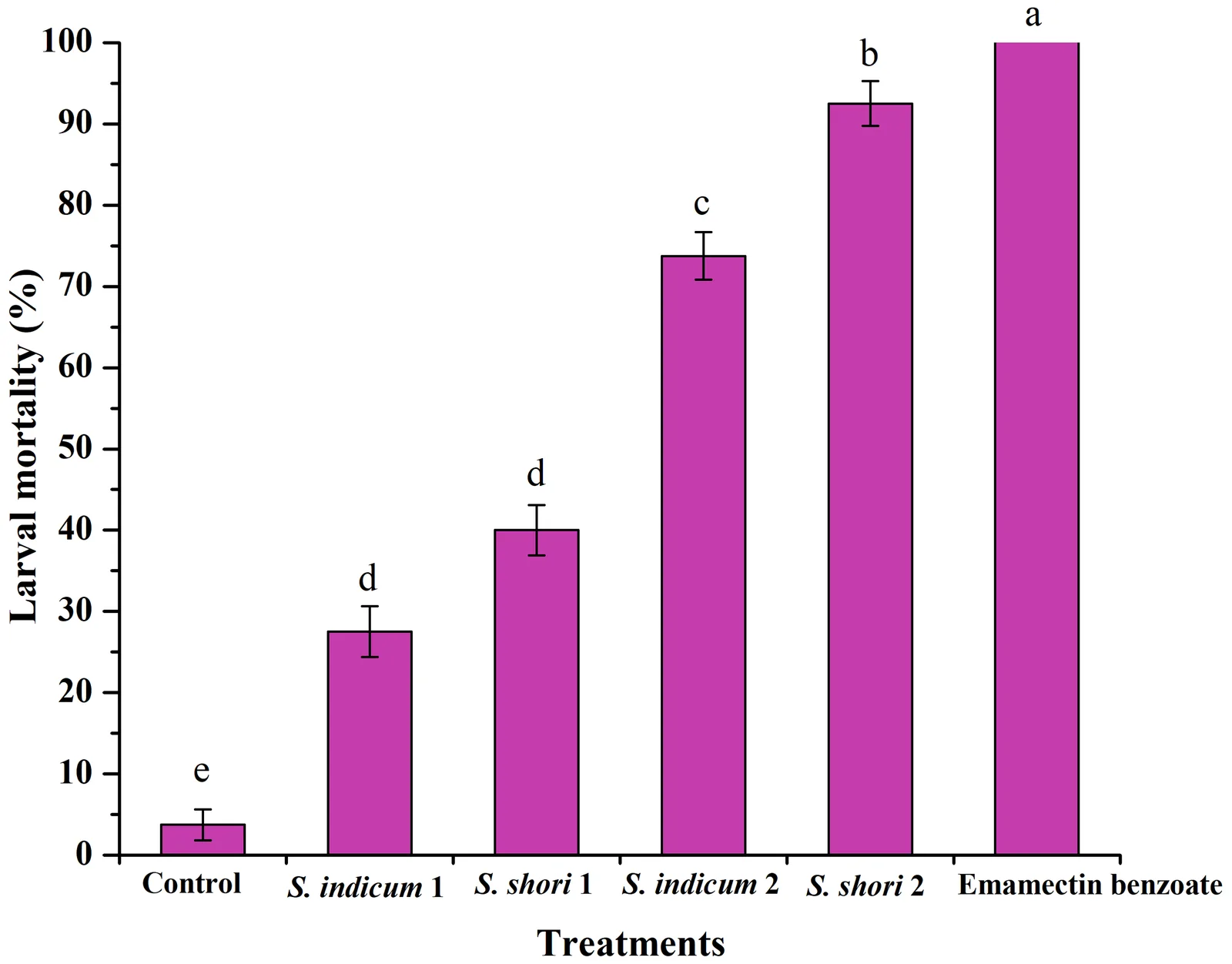
Mean percent mortality of fourth-instar larvae of *Spodoptera frugiperda* ± SE in pot experiments with different treatments at 5 days after application. Bars with different letters indicate significant differences for different treatments (P < 0.05, Tukey's test). 1 = 500 IJs larva^−1^ and 2 = 1000 IJs larva^−1^. Emamectin benzoate was used at a rate of 12.5 g a.i. ha^−1^.

Five days after treatment, larval mortality of *S. frugiperda* differed significantly among the tested treatments (F_5, 45_ = 20.04; P < 0.0001). The percentage of mortality was significantly higher (100.0%) in plants treated with emamectin benzoate compared to those treated with nematode species. Results showed that a larval mortality rate of 92.5% in maize plants treated with *S. shori* NBAIRS80 at a dosage of 4000 IJs plant^−1^ (1000 IJs larva^−1^), while plants treated with *S. indicum* NBAIRS58 at the same rate of 4000 IJs plant^−1^ (1000 IJs larva^−1^) showed a mortality rate of 73.7%. Among the nematode species, *S. shori* NBAIRS80 resulted in significantly higher mortality than *S. indicum* NBAIRS58. A larval mortality rate of 3.7% in was recorded in untreated control plants (**Figure 7**).

### 3.6 Field experiment

**Figure 8:**
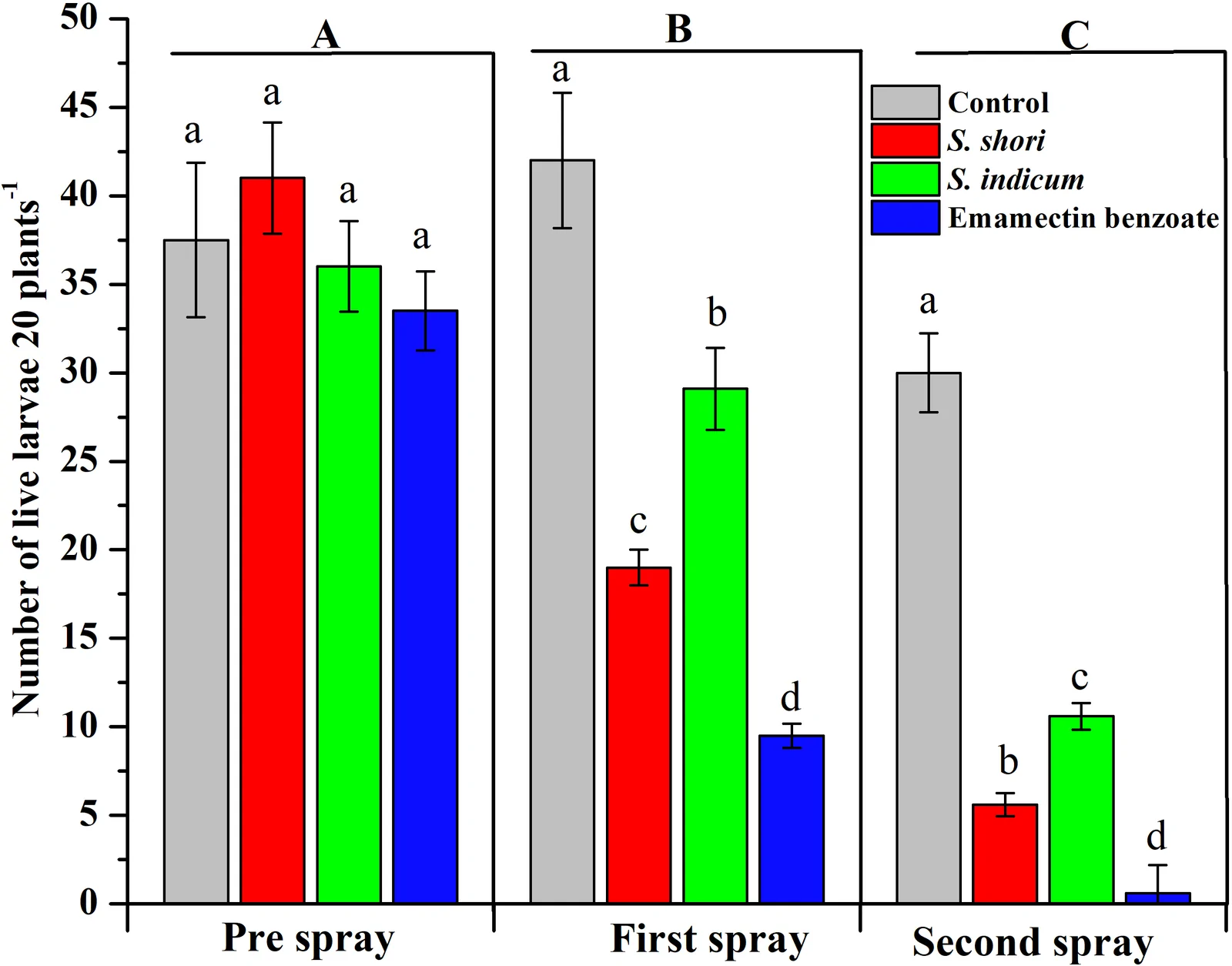
Mean number of *Spodoptera frugiperda* larvae ± SE on 20 maize plants with different treatments at 7 days after application in a naturally infested maize field at Bommanahalli village in Chikkaballapura taluk and district, Karnataka, India, during the Rabi season. Both EPN species were applied at a concentration of 2.5 × 108 IJs ha^−1^. Emamectin benzoate was sprayed at a rate of 12.5 g a.i. ha^−1^ in both sprays. Bars with different lowercase alphabets on top of the error bars depict a statistically significant difference between the treatments, and alphabets with uppercase letters denote a significant difference between the sprays (P < 0.05, Tukey's test).

At the start of the experiment, larval counts per 20 plants did not differ significantly among treatments (F_3, 36_ = 1.08; P = 0.368). Application of EPNs and emamectin benzoate resulted in a significant reduction in the number of live larvae during both the first (F_3, 36_ = 47.93; P < 0.0001) and second (F_3, 36_ = 79.61; P < 0.0001) spray rounds. Following the second application, the lowest larval density (0.6 ± 1.6 larvae 20 plants^−1^) was recorded in plots treated with emamectin benzoate. A significantly higher larval density was recorded in the untreated control, where the population reached 30 ± 2.24 larvae plants^−1^. Among the EPN treatments, plots treated with *S. shori* at 2.5 × 10^8^ IJs ha^−1^ recorded 5.6 ± 0.65 larvae 20 plants^−1^, whereas *S. indicum* at the same rate resulted in 10.6 ± 0.75 larvae 20 plants^−1^ after the second spray (**Figure 8**).

## 4 Discussion

The use of entomopathogenic nematodes (EPNs) to combat insect pests has been effectively showcased worldwide. This study highlights the efficacy of the recently discovered EPN species, *Steinernema shori* NBAIRS80 and *S. indicum* NBAIRS58, in attacking different larval stages and pupae of *S. frugiperda* in both laboratory and field settings. Additionally, it investigates the reproductive capabilities of these EPN species on *S. frugiperda* larvae. In both the Petri dish and soil column assays, the lowest concentration tested (20 IJs larva^-1^) resulted in low mortality rates for both *S. shori* and *S. indicum* against third- and sixth-instar larvae. However, third-instar larvae exhibited greater susceptibility to nematode infection than sixth-instar larvae under these treatment conditions. Findings from both assays indicated that the virulence of EPN species not only dependent on their IJs concentration but also developmental stages of host insect. Consistent with the present findings, several studies have reported that third instar larvae of *S. frugiperda* are generally more susceptible to EPNs than sixth instars. Acharya et al. (2020) recorded 62.0% mortality in third-instar larvae compared with 56.0% mortality in sixth-instar larvae following treatment with *H. bacteriophora* at 50 IJs larva^-1^, 72 h after inoculation. Similarly, Wang et al. (2022) reported mortality rates of 84.0% and 56.0% in third- and sixth-instar larvae, respectively, after exposure to *Heterorhabditis* sp. strain 22835 at 150 IJs larva^-1^, 60 h after treatment. Patil et al. (2022) also found higher susceptibility in younger larvae, recording 34.0% mortality in third-instar larvae and 24.0% mortality in fourth-instar larvae treated with *H. indica* at 25 IJs larva^-1^, five days after treatment. Likewise, Mohamed and Shairra (2023) reported 44.4% mortality in third-instar larvae, whereas sixth-instar larvae showed no mortality following inoculation with *S. carpocapsae* at 150 IJs larva^-1^, 48 h after treatment. In addition, Rashed et al. (2024) recorded 80.0% mortality in third-instar larvae compared with 60.0% mortality in sixth-instar larvae when exposed to *S. carpocapsae* at 50 IJs larva^-1^, 96 h after treatment.

The variation in susceptibility recorded among the instars may result from a combination of morphological, physiological, and immunological differences throughout the developmental stages. Early instars typically exhibit weaker immune responses and thinner cuticles (Brodeur and Vet, 1995; Gillespie et al., 1997), which facilitate the penetration and establishment of EPNs. Previous studies have shown that immune-related parameters increase with larval development. Jalali and Salahi (2008) reported that, in *Papilio demoleus*, fourth-instar larvae exhibited higher total hemocyte counts (THC) (9244.0 mm^-^³) than third-instar larvae (3872.0 mm^-3^) [36]. Similarly, Valadez-Lira et al. (2012) found that, in *Heliothis virescens*, phenoloxidase activity was lower in second-instar larvae (0.38 mg mL^-1^) than in fourth-instar larvae (1.45 mg mL^-1^). They also reported lower total protein levels in second-instar larvae (1.42 ± 0.38 mg mL^-1^) compared with fourth-instar larvae (1.99 ± 0.38 mg mL^-1^) Valadez-Lira et al. (2012). Since hemocytes, phenoloxidase, and hemolymph proteins play essential roles in both cellular and humoral immunity, the comparatively lower levels of these components in younger larvae may contribute to their reduced capacity to defend against microbial pathogens. Consequently, we hypothesize that the invading IJs may have successfully bypassed the defense mechanisms of third instar larvae, resulting in higher mortality rates compared to fourth and sixth instar larvae, even at lower concentrations. In our investigation, the body size of the host insect may not have been a significant factor, as third instar larvae weighed 37.6 ± 0.32 mg. In this size range, IJs can easily penetrate the host body; otherwise, the third instar larvae could have posed challenges for the penetration of IJs, leading to lower susceptibility to the EPN species.

Both species resulted in decreased mortality rates in pupae in contrast to larvae. The reduced vulnerability of pupae to EPNs in comparison to larval stages can be attributed to their biological traits. Pupae usually do not feed, are encased in a tough cuticle, and exhibit diminished metabolic activity, which restricts nematode entry and bacterial growth. Kaya and Gaugler (1993) found that the pupal stages of insects are generally less vulnerable to EPNs due to fewer entry points and restricted physiological activity, which closely corresponds with the diminished mortality seen at lower IJ concentrations in this research. Likewise, Grewal et al. (2005) recorded that higher IJ concentrations are generally needed to cause notable mortality in less susceptible host stages like pupae. These findings emphasize that while the pupae of *S. frugiperda* are somewhat more resilient than the larval stages, effective control can still be achieved with higher IJ concentrations. From a practical perspective, focusing on the pupal stage within soil environments might aid in decreasing adult emergence and the resulting population growth. However, the requirement for higher IJ concentrations could influence cost-effectiveness and methods of field usage. Consequently, integrating EPN applications with additional management tactics may enhance pest control efficiency and increase sustainability in field conditions.

The current research shows that both *S. shori* and *S. indicum* effectively invaded and multiplied within the hemocoel of third-instar *S. frugiperda* larvae, validating their pathogenic capacity. Nevertheless, penetration efficiency and reproductive output showed considerable variation with IJ concentration and between nematode species, highlighting complex density-dependent interactions during host infection. Initially, penetration rates increased with increasing concentrations of IJ, but they decreased at higher doses, especially beyond 300 IJs larva^-1^ for *S*. *shori* and 400 IJs larva^-1^ for *S. indicum*. Similarly, Kary et al. (2022) reported that a negative correlation between penetration rate and IJ concentration in the potato tuber moth, *Phthorimaea operculella*. Furthermore, it can be concluded that the penetration rate increases with IJ concentration to a certain optimal threshold, beyond which the penetration rate may start to decline. This decline at increased IJ densities could be due to intraspecific competition, interference among IJs, or host immune responses activated by an overload of nematodes Kaya and Gaugler (1993). Additionally, Stock and Goodrich-Blair (2012) emphasized that high IJ densities may lead to diminished infection efficiency because of limited penetration sites and increased antagonistic interactions among invading IJs.

The present findings from pot and field experiment demonstrate that larval mortality of *S. frugiperda* in maize varied markedly among treatments, with a clear superiority of chemical control over biological agents under the tested conditions. Nevertheless, this reduced larval mortality by EPNs can be compensated by their environmental safety, host specificity, and compatibility with other control methods. The significantly higher efficacy of *S. shori* than *S. indicum* suggests greater virulence and adaptability under plant conditions, possibly due to improved host-seeking behavior, better foliar persistence, or more efficient infection once inside the host. These results align with the findings of Grewal et al. (2005), who stated that differences in virulence among EPN species are often linked to their environmental tolerance, and symbiotic bacterial efficacy. The significant increase in larval mortality after both the first and second round of sprayings suggests that repeated applications enhance nematode persistence and infection pressure, thereby improving control efficacy. One possible limitation of the pot experiment is the risk of cannibalism among the four fourth-instar *S. frugiperda* larvae placed in each maize whorl. Cannibalistic behavior is well established in this species, especially during the later larval stages, and may affect larval survival independently of EPN infection (Chapman et al., 1999; Andow et al., 2015). Since the same larval density was maintained across all treatments, any mortality due to cannibalism was anticipated to be similarly distributed among treatments, thus reducing its impact on the comparative evaluation of EPN efficacy. Nevertheless, it cannot be entirely ruled out that cannibalism played a role in the observed mortality or variability in the experiment. Future research utilizing individually confined larvae or direct behavioral observations would yield a more accurate assessment of mortality specifically due to EPN infection.

## 5 Conclusion

In conclusion, both species of the tested EPNs successfully invaded the host and completed their life cycle, resulting in a significant production of IJs, which demonstrates a strong reproductive capacity and the ability to persist through recycling. However, *S. shori* exhibits remarkable potential as a biocontrol agent for *S. frugiperda* due to its superior efficacy and reproductive capabilities. *Steinernema shori* significantly reduced pest populations under both pot and field conditions compared to *S. indicum*. Future studies should concentrate on extensive field evaluations across various agro-climatic conditions and with other insect pest species to confirm its effectiveness. Furthermore, compatibility with other elements of integrated pest management, such as agrochemicals and natural enemies, needs to be evaluated. Research on environmental tolerance and long-term soil persistence will further support its commercialization and sustainable use in pest management programs.

## Data Availability

The raw data supporting the conclusions of this article will be made available by the authors, without undue reservation.
